# Catalytic asymmetric acylation reactions using isothiourea catalysts: a Decade's update

**DOI:** 10.1039/d6ra01841f

**Published:** 2026-04-22

**Authors:** Sushree Ahalya Khatua, Riya Ghosh, Amit Kumar Simlandy

**Affiliations:** a Department of Chemical Sciences, Indian Institute of Science Education and Research Berhampur Berhampur 760003 India asimlandy@iiserbpr.ac.in

## Abstract

Acyl transfer reactions have gained significant research attention as crucial asymmetric transformations in organic synthesis over the past few decades. Among various chiral nucleophilic catalysts, the chiral isothiourea catalysts, first introduced by Birman in 2006, have emerged as remarkably effective tools for achieving high enantioselectivity in acylation processes. Recent advances in density functional theory (DFT) calculations have elucidated the underlying mechanisms, revealing that S⋯O isothiouronium interactions between the catalyst and the acyl group, along with other non-covalent interactions, play a vital role in stabilizing the transition state. This review presents a comprehensive documentation of isothiourea-catalyzed enantioselective acylation reactions across a diverse array of alcohols and amines, outlining their methodologies, such as kinetic resolution, dynamic kinetic resolution, and atroposelective approaches. Additionally, we discuss the pertinent stereochemical models and highlight the synthetic utility of the resulting enantioenriched products, illustrating the broad applications of this catalytic strategy.

## Abstract 2

This comprehensive review highlights the isothiourea-catalyzed enantioselective acyl transfer reactions developed over the last decade.

## Graphical abstract

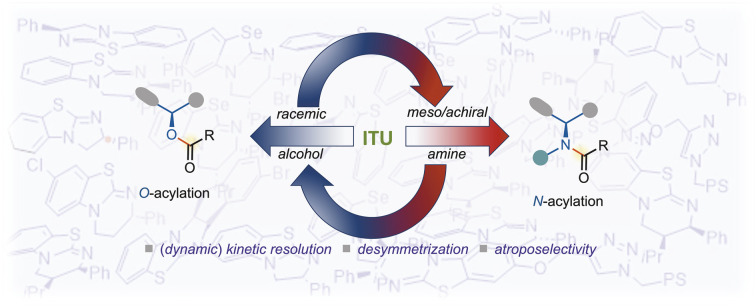

## Introduction

Acyl transfer represents a pivotal transformation in organic synthesis, functioning not only as a protective strategy for various functional groups, including alcohols, amines, and thiols,^1^ but also facilitating the formation of essential products, such as esters, amides, and thioacetals. The prevalence of acylated molecules, particularly esters in natural products,^2^ coupled with the critical role of amides in protein structure^3^ and function, underscores the importance of acyl transfer in chemical biology and medicinal chemistry. A key application of acyl transfer is in the catalytic asymmetric acylation reactions, which have proven particularly useful for the synthesis of biologically relevant molecules, natural products, and pharmaceuticals. These acyl-transfer reactions are typically catalyzed by nucleophilic catalysts that facilitate the effective transfer of acyl groups. The most commonly used catalysts in this process are *N*-heterocyclic carbenes (NHCs),^4^ imidazole derivatives,^5^ chiral 4-dimethylaminopyridine (DMAP),^6^ pyridinium ylide (PPY),^7^ various chiral phosphines,^8^ and isothiourea catalysts (C).^9^ All of these catalyst classes possess unique characteristics that can improve the selectivity and efficiency of the acylation process. Isothiourea catalysts exhibit greater nucleophilicity than traditional catalysts (*i.e.*, PPY and phosphines) and demonstrate a similar nucleophilicity to DMAP.^10^ In acyl transfer reactions with isothiourea, the stabilization of the key acyl ammonium intermediate through various secondary interactions results in exceptional stereocontrol, unlike with the DMAP catalyst where the loss of aromaticity of the pyridine ring occurs.^12^ Furthermore, the ease of preparation, bench stability, and convenient handling enhance the appeal of isothiourea catalysts as a preferred choice for asymmetric catalysis. A handful of literature reviews have previously addressed asymmetric transformations facilitated by these Lewis base catalysts. Bressy's review article covered the development, properties, and structures of isothiourea catalysts, and the different modes of catalyst activation for enantioselective transformations up to 2016.^13^ There have been significant developments in catalytic enantioselective asymmetric transformations over the last decade. In this review, we concentrate solely on the isothiourea-catalyzed asymmetric acylation *via O*- and *N*-acylation. We will provide a comprehensive exploration of the reaction mechanisms underpinning the isothiourea-catalyzed acylation, highlighting the stereochemical models that lead to the formation of a particular enantiomer. The evolution of other isothiourea analogues and their application in the synthesis of important biologically relevant molecules and drugs will also be discussed.

**Fig. 1:**
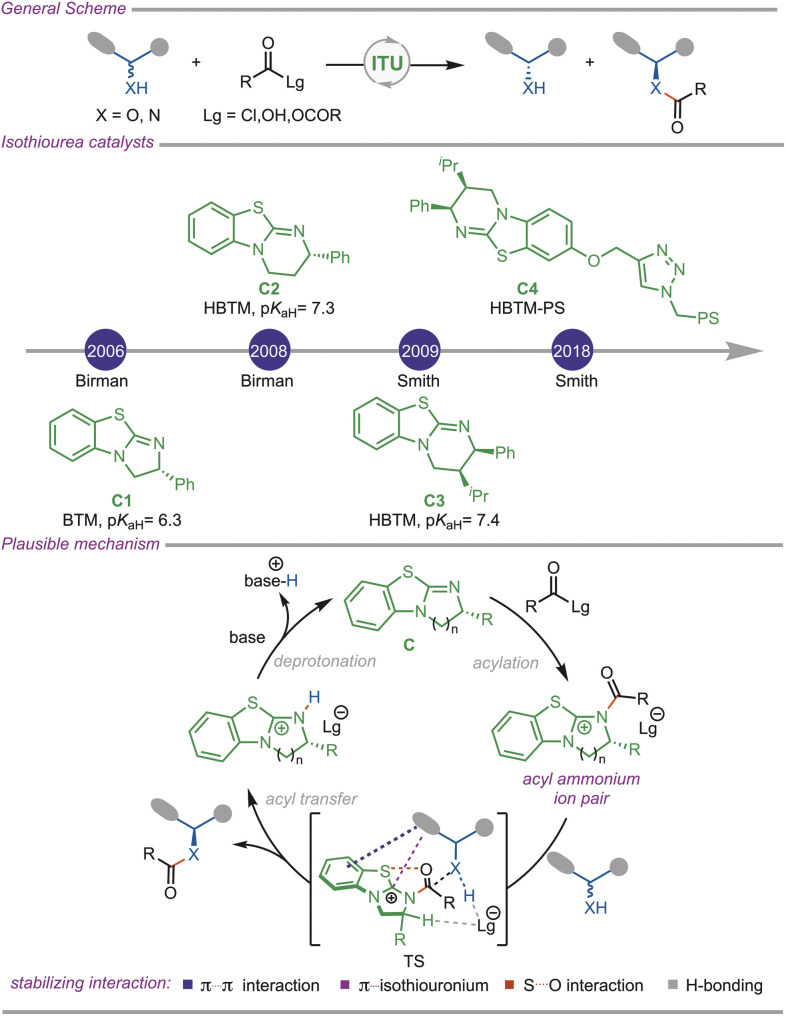
General representation of isothiourea catalysis (p*K*_aH_ in DMSO).^11^

Isothiourea organocatalysts have garnered considerable attention in asymmetric catalysis due to their remarkable ability to facilitate diverse chemical transformations. Notably, the asymmetric acylation of secondary alcohols with acylating agents using the isothiourea catalyst C1 was first reported by Birman in 2006.^9^ This pioneering work has advanced the application of isothiourea-based catalysts in asymmetric catalysis, significantly expanding their utility. In 2008, Birman and Xi modified the non-aromatic ring of the BTM catalyst by adding substituents to synthesize HBTM (C2) and HTM catalysts, which exhibited excellent selectivity and reactivity^14^ (Fig. 1). Subsequently, a series of HBTM (C3) analogues was developed by researchers including Birman,^15^ Smith,^16^ Shiina,^17,18^ Gröger,^19^ and Okamoto.^20^ The introduction of additional substituents in these analogues altered their conformation, enhanced noncovalent interactions, and stabilized the transition state. In 2018, Smith developed a polymer-based HBTM (C4) catalyst that demonstrated excellent selectivity and exhibited recyclability.^21^

These Lewis base catalysts are highly nucleophilic in nature and readily react with the acyl donor to form the acyl ammonium ion pair, where the S-atom of the catalyst interacts with the oxygen atom of the acyl functionality *via n*(O) → σ*(S) to control the spatial orientation of the catalyst.^22,23^ Common acylating agents include acyl chlorides and anhydrides. This highly electrophilic acyl intermediate readily reacts with diverse heteroatom-based nucleophiles, and the transition state leading to the product is stabilized *via* several non-covalent interactions. Hydrogen bonding^24^ of the counter anion with both the acidic proton of the nucleophile and the catalyst C–H bond, van der Waals forces, π⋯π stacking interactions, C–H/π interactions, and electrostatic interactions between the positive charge on the catalyst with the electron-rich π-system of the nucleophile allow for the precise alignment of reactants, facilitating the desired reaction pathway and helping to achieve excellent enantiocontrol.^25^ After releasing the product, the catalyst forms an ion pair with the acyl-donor counteranion, which undergoes base-mediated deprotonation to regenerate the catalyst for the next cycle. Among the different heteroatoms, *O*-acylation has been explored extensively.

### *O*-acylation

The *O*-acylation process, facilitated by the isothiourea catalyst, has emerged as a powerful method for the kinetic resolution and dynamic kinetic resolution (DKR) of racemic alcohols and the desymmetrization of *meso*-diols. Diverse alcohols, when treated under isothiourea-catalyzed acylation conditions, form an ester that can be easily converted into various functional groups and smoothly translated into value-added products *via* simple steps.^13^

**Scheme 1:**
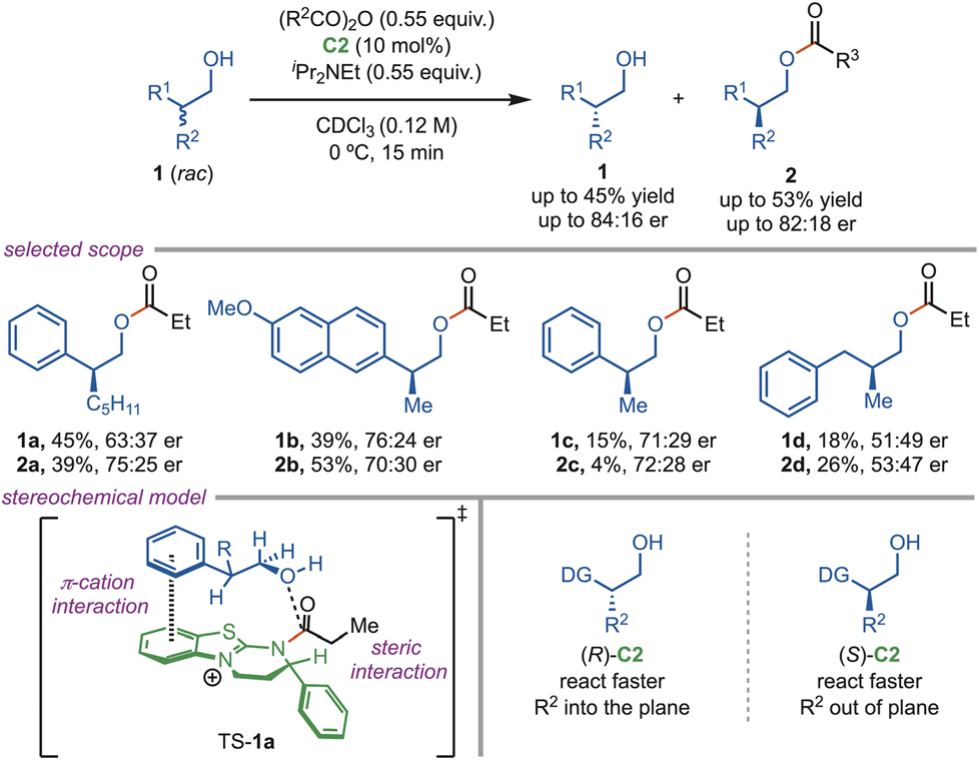
Determination of the absolute configuration of β-primary alcohols.

In 2017, Rychnovsky and coworkers reported a methodology for the determination of the absolute configuration of β-chiral primary alcohols (1) using the competing enantioselective conversion (CEC) approach *via* the C2 enantioselective acylation reaction (Scheme 1). The success of this protocol largely depended on the presence of a directing group, such as a (hetero)-arene or an enone, at the stereogenic centre. The optically active alcohol was reacted in parallel with either the (*R*)-C2 or the (*S*)-C2 catalyst using propionic anhydride and ^*i*^Pr_2_NEt as the base. Generally, (*S*)-C2 reacts faster with heteroaryl substituents, and it depends on the substrate stereochemistry whether it is out of plane or in the plane.^26^ It is proposed that the π–cation interaction helps stabilize TS-1a, leading to the product.

**Scheme 2:**
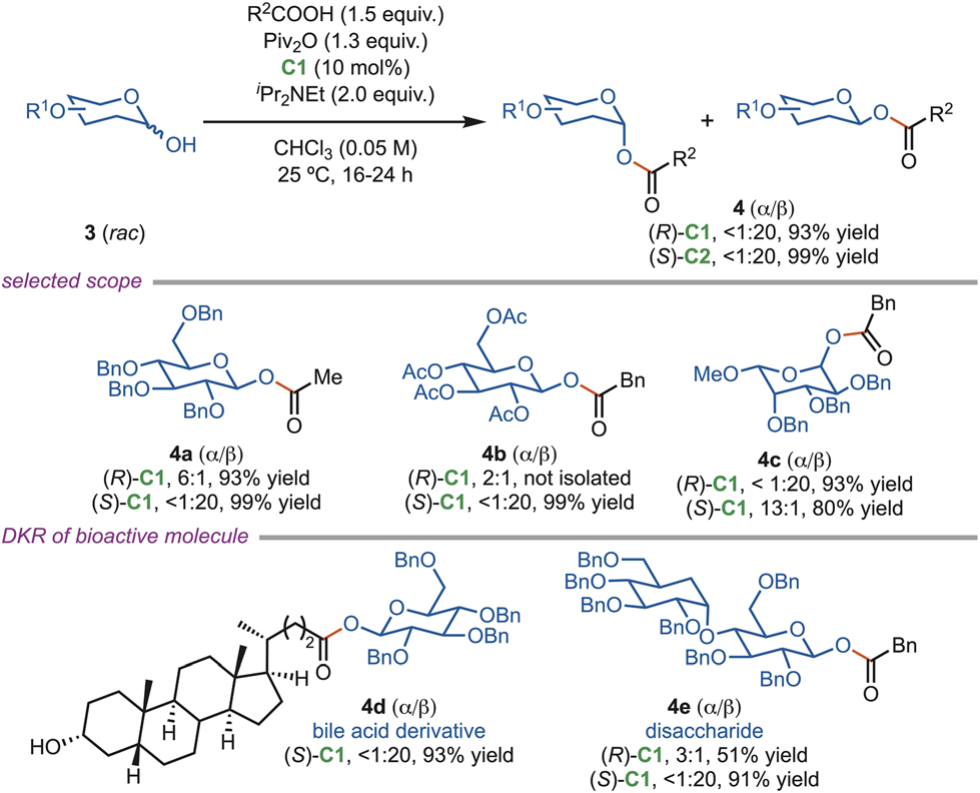
Catalyst-directed acylative DKR of anomeric hydroxyl groups.

In 2017, Tang *et al.* developed a chiral isothiourea-directed dynamic kinetic diastereoselective acylation (DKDA) strategy that enabled the selective synthesis of either α- or β-anomeric esters from various sugar derivatives (Scheme 2). A broad scope of acylating acid compounds, including simple and drug-derived ones, was successfully introduced, with a general preference towards the formation of β-anomeric products with the (*S*)-C1 catalyst. The α-anomeric esters were obtained in moderate selectivity using the (*R*)-C1 catalyst. The α-anomeric esters were smoothly reduced to the corresponding ethers without stereochemical scrambling. In contrast, the β-anomeric esters followed an alternative reaction pathway, leading to the formation of an acetyl ester. This methodology was further applied to the synthesis of β-glucose acyl conjugates with high selectivity that target cancer cells.^27^

**Scheme 3:**
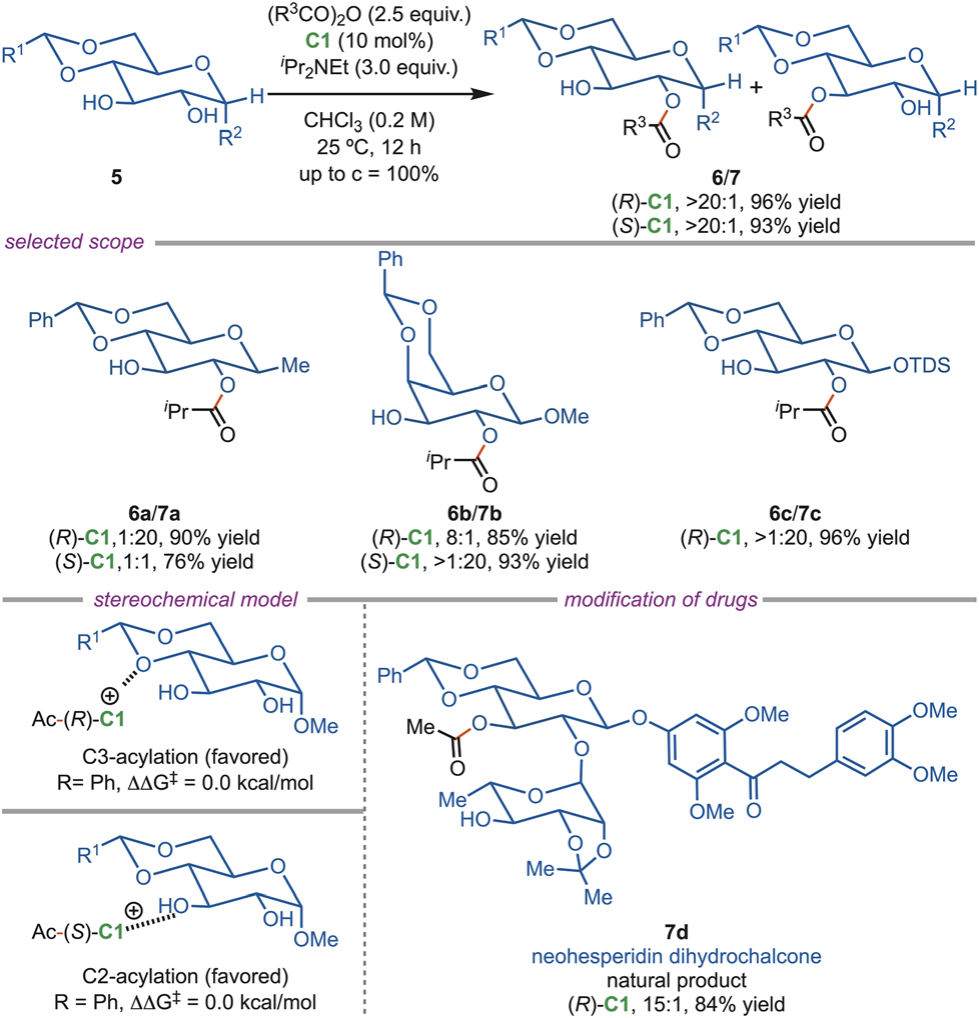
Site-selective acylation of pyranoses.

In a separate study reported in 2017, Tang *et al.* developed a site-selective acylation protocol for pyranoses using an isothiourea catalyst, enabling the C-3 *vs.* C-2 selectivity (Scheme 3). DFT studies revealed that the observed site selectivity originates from stabilizing cation–π interactions between the C1-catalyst and the sugar substrate (5), which are influenced by the relative positions of the hydroxyl groups. These interactions depend on the substrate stereochemistry and are favored for equatorial –OH/–OR groups, which are devoid of 1,3-syndiaxial interactions. It was observed that (*R*)-C1 catalyst favors C3-acylation by stabilizing cation-n interactions, whereas the (*S*)-C2 catalyst shifts preference toward the C-2 position when the C-3 lone pair is sterically or electronically blocked. This strategy was successfully extended to disaccharides and complex sugar derivatives, including trehalose and neohesperidin dihydrochalcone (7d), demonstrating its synthetic utility for late-stage functionalization.^28^

**Scheme 4:**
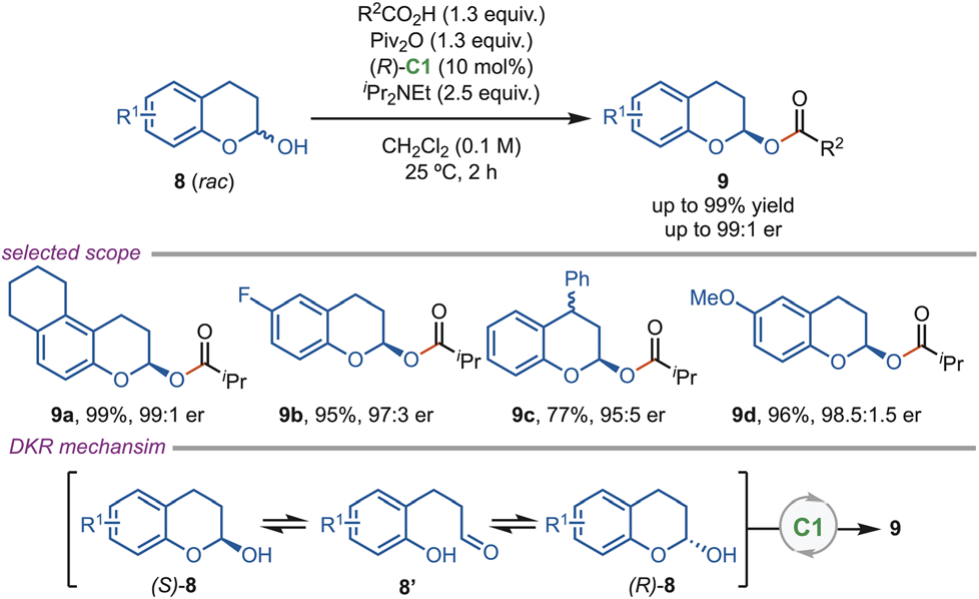
Dynamic kinetic enantioselective acylation of 2-chromanols.

In the following year, a C1-catalyzed dynamic kinetic enantioselective acylation of racemic 2-chromanol (8) was reported by the Tang group (Scheme 4). DFT studies revealed that the enantioselectivity was controlled by cation-n interactions between chromanol and catalyst (C1) in the transition state (Scheme 4). The generality of this protocol was evaluated by installing both electron-releasing and electron-deficient substituents, which led to the formation of the product (9) with excellent yield and enantioselectivity. The antipode of the product was also obtained using the (*S*)-C1 catalyst.^29^

**Scheme 5:**
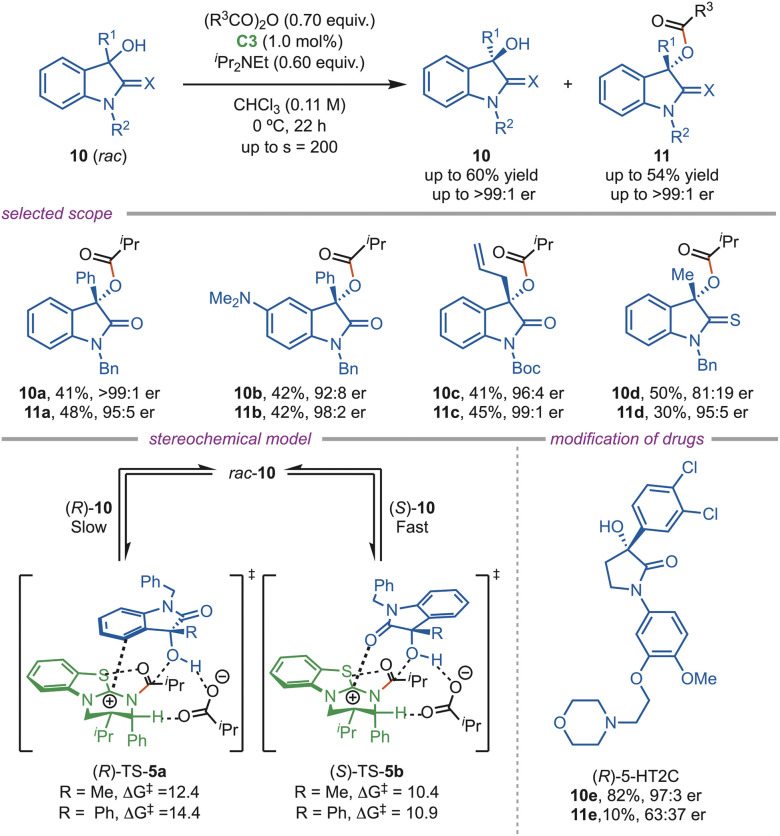
Acylation of tertiary heterocyclic alcohols.

In 2018, Smith *et al.* disclosed a synthetic strategy for the acylative kinetic resolution of the sterically hindered tertiary heterocyclic alcohol (10). The C3-catalyst showed high efficiency, delivering a selectivity factor of up to 200 at 1.0 mol% catalyst loading (Scheme 5). The reaction tolerated diverse aryl, alkyl, and heteroaryl substituents, affording excellent enantioselectivity. However, sterically hindered groups, such as alkyne substituents and *tert*-butyl substrates, showed poor enantioselectivity. DFT studies showed that (*R*)-TS-5a was destabilized by π⋯isothiouronium interaction. This group successfully developed a methodology for the synthesis of the 5-HT2C (10e) antagonist, yielding both enantiomers efficiently.^30^

**Scheme 6:**
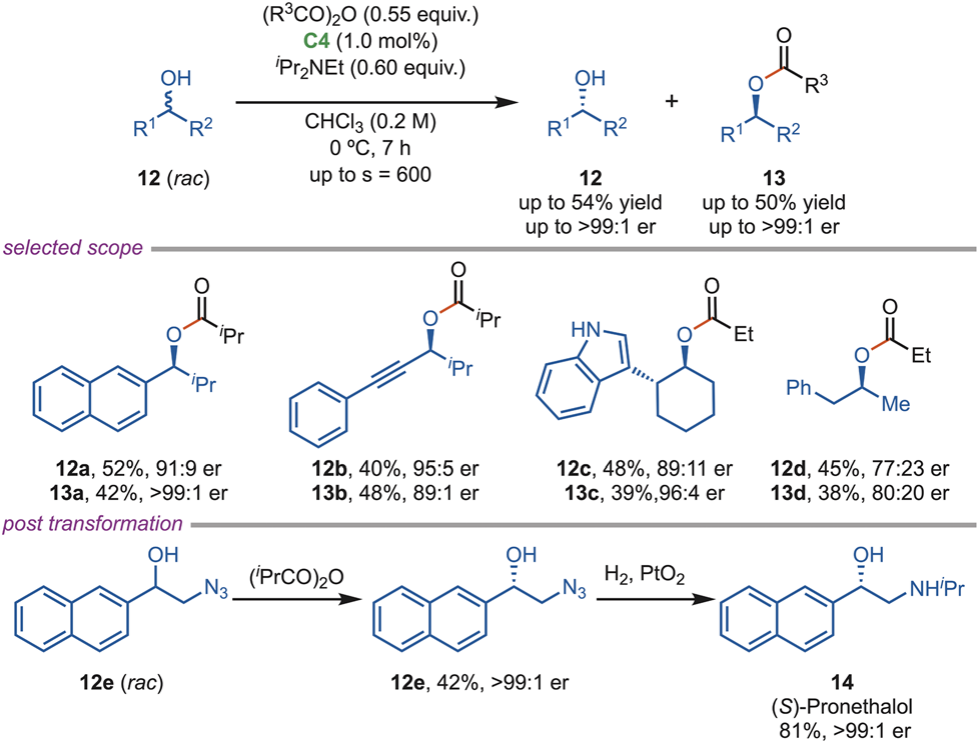
Acylative kinetic resolution of secondary alcohols using polymer-based HBTM.

In the same year, the Smith group reported the first example of acylative kinetic resolution of racemic secondary alcohols 12 using a recyclable polymer-based immobilized C4 catalyst, implemented under both batch and continuous-flow conditions (Scheme 6). A polymer-supported isothiourea catalyst was designed to achieve high enantioselectivity with excellent recyclability. For the batch reactions, an optimal catalyst loading of 1.0 mol% was identified, delivering selectivity factors of up to 600. Secondary alcohols having both electron-donating and electron-withdrawing substituents were readily accommodated, although these substrates generally exhibited lower selectivity factors. In contrast, propargylic and allylic alcohols showed good enantioselectivity. Notably, the kinetic resolution of both *cis*- and *trans*-phenylcyclohexanol was successfully achieved. The synthetic utility of this approach was highlighted through its application to the enantioselective synthesis of (*S*)-pronethalol (14), a β-blocker used in the treatment of cardiac arrhythmias and coronary heart disease. Furthermore, this protocol demonstrated potential for the preparation of enantiopure 1,2-amino alcohols. Continuous-flow systems demonstrated efficient catalyst recycling and scalability, underscoring the potential of this strategy for industrial applications.^21^

**Scheme 7:**
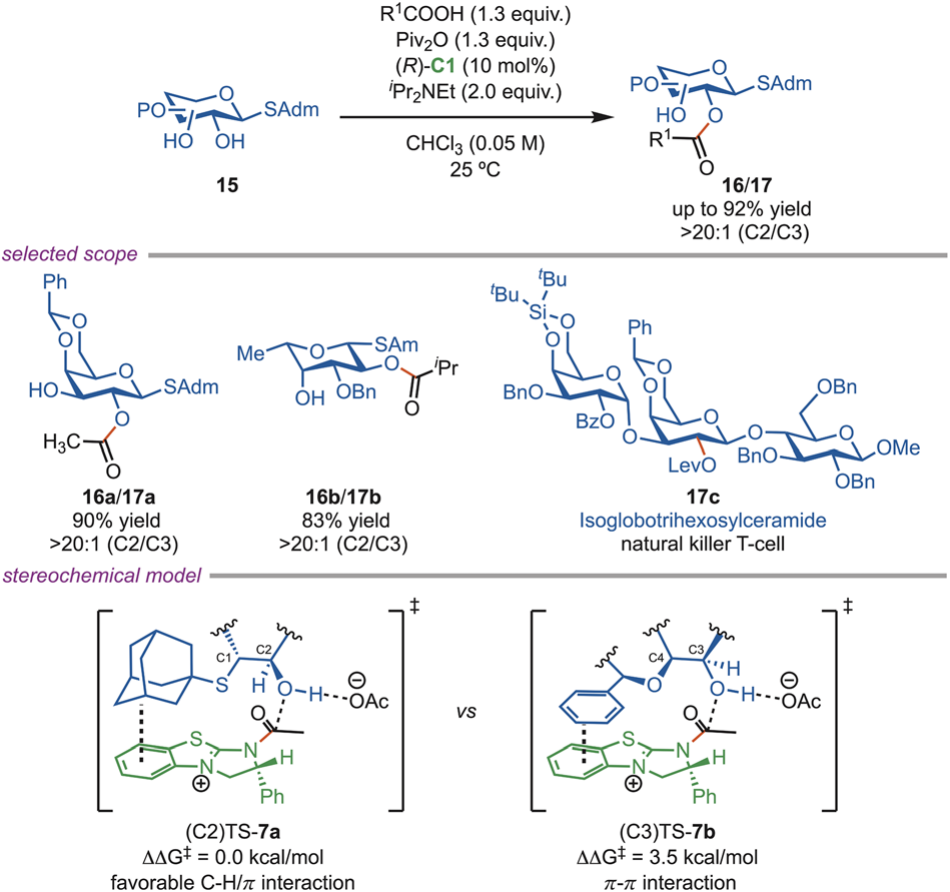
Site-selective acylation of *S*-glycosides.

The Tang group further demonstrated that an *S*-adamantyl group can effectively direct the C-2 site-selective acylation of *S*-glycosides using an (*R*)-C1 catalyst (Scheme 7). This method achieved excellent selectivity (>20 : 1) across a wide variety of glycoside substrates, including diols, triols, and tetrols. In cases where the adamantyl group obstructs access to the C-2 hydroxyl group, the acylation was redirected to the C-3 position instead. DFT studies revealed that the C–H⋯π interaction between the adamantyl C–H bonds and the catalyst aromatic ring stabilized the (C-2)-transition state (TS-7a), making it more favorable. Notably, this approach enabled the conversion of selectively acylated *S*-glycosides into activated glycosyl donors, facilitating the efficient assembly of oligosaccharides. The synthetic utility of this strategy was illustrated by the synthesis of isoglobotrihexosylceramide, an antigen for natural killer T cells (17c).^31^

**Scheme 8:**
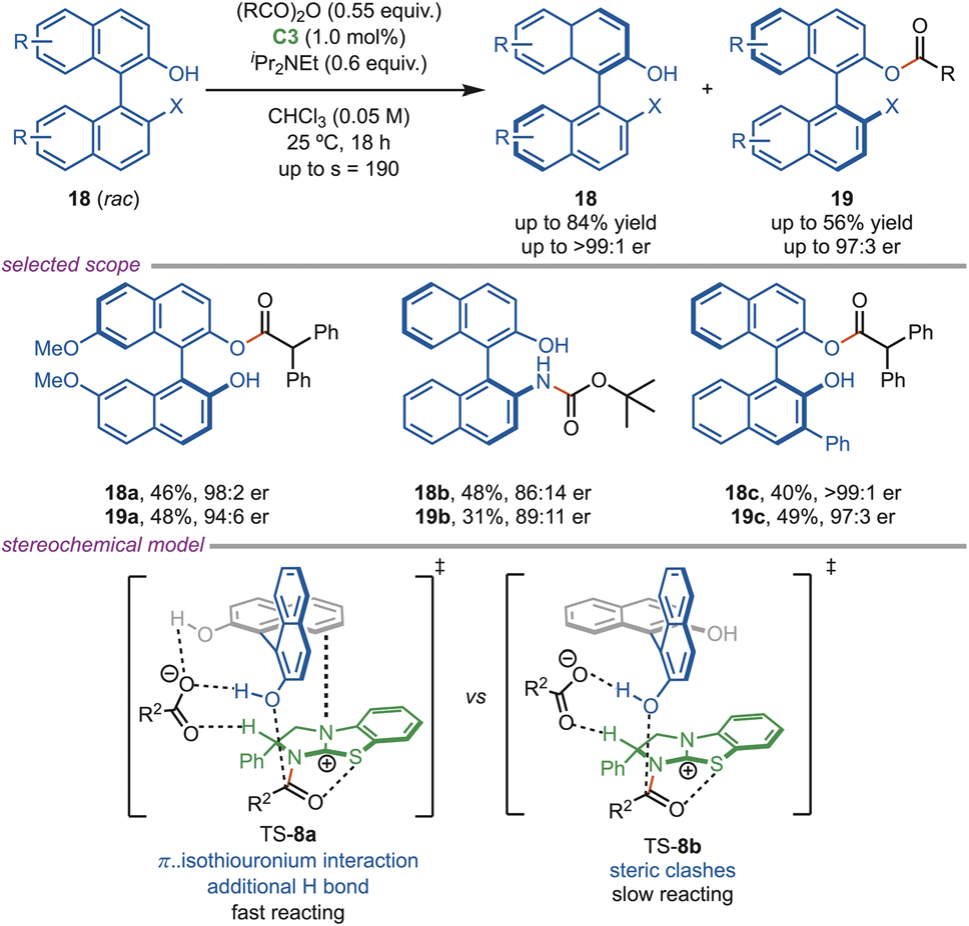
Acylative kinetic resolution of BINOL using HBTM.

The axially chiral biaryl compounds are found in a wide range of bioactive molecules and pharmaceuticals, and they are also used as ligand precursors for diverse catalytic asymmetric transformations (Scheme 8). Therefore, the selective functionalization of biaryl diols, particularly through acylative kinetic resolution, has attracted sustained interest. An early example was reported by Sibi, who demonstrated the mono-acylation of biaryl diols using a fluxionally chiral DMAP-based catalyst, achieving moderate to good selectivity factors.^32^ In 2014, Zhao *et al.* reported a method for acylative kinetic resolution of an axially chiral biaryl diol using NHC catalysis.^33^ In 2019, Smith *et al.* developed a regio- and atropo-selective acylative kinetic resolution of a biaryl diol using an isothiourea catalyst (C3). During this acylative transformation, the formation of a small amount of diester product was observed along with the desired monoester (19). A control experiment shows that the presence of two hydrogen-bond donors is required to achieve both high reactivity and good selectivity. The proposed transition state features dual hydrogen-bonding interactions between the isothiourea-acyl ammonium intermediate and the diol, which enforce both the regio- and enantio-selectivity. The π–π interactions between the catalyst and the biaryl framework provide additional stabilization to TS-8a. Moreover, the NOBIN derivative (19B), where both the amine and the alcohol are present, showed excellent conversion and selectivity.^34^

**Scheme 9:**
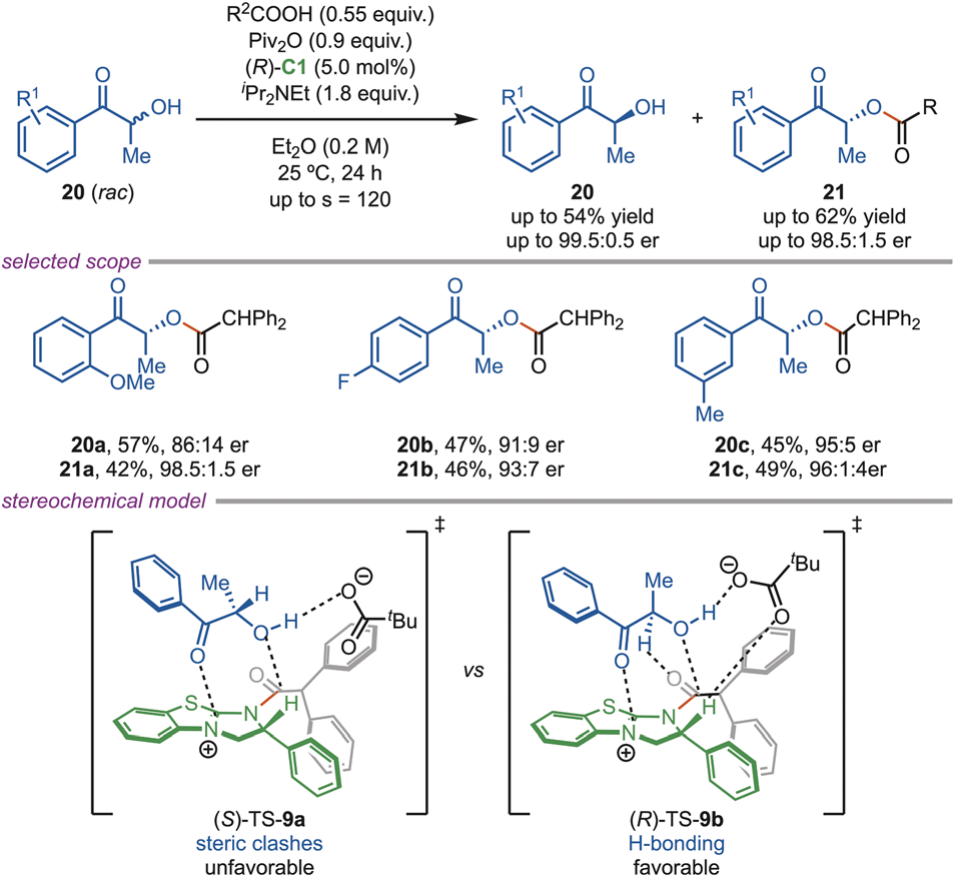
Acylation of hydroxyaryl ketones.

In 2020, the Shiina group disclosed a (*R*)-C1-catalyzed kinetic resolution of racemic 2-hydroxyarylketones (20), yielding the product with a high enantiomeric ratio (Scheme 9). This study showed that the addition of a tertiary amine (^*i*^Pr_2_NEt) as a base did not significantly improve selectivity (*s*-values). Screening of various acyl donors revealed that the sterically bulky donor produced the desired products with higher enantioselectivity. Further, 2-hydroxyarylketones bearing *ortho*-, *meta*-, and *para*-substituents with varying electronic properties underwent this transformation smoothly, affording the ester (21) with high *s*-values. The DFT calculation showed that the favorable (*R*)-TS-9b involves a hydrogen-bonding interaction between the hydroxyl group and pivalate, plus coordination between the ketone carbonyl and the thiouronium cation.^35^

**Scheme 10:**
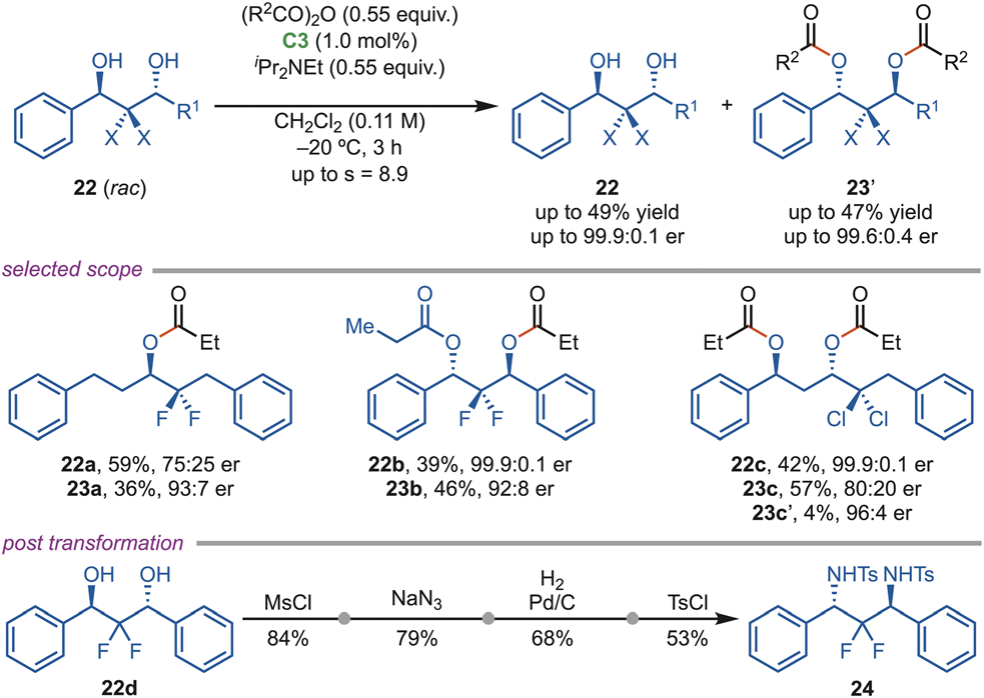
Acylative kinetic resolution of α,α-difluorohydrin.

In 2015, the Bressy group developed a highly enantioselective isothiourea (C3) catalyzed acylative desymmetrization of acyclic *meso*-1,3-diol.^36^ In late 2021, the Bressy group reported an enantioselective acylative kinetic resolution of α,α-difluorohydrins (*rac*-22), providing access to enantio-enriched organofluorine compounds (23), which are of significant relevance to the pharmaceutical and agrochemical industry (Scheme 10). The difluoromethylene group (C(sp^3^)F_2_)-containing diols showed a strong electrostatic fluorine–cation interaction, which enhanced the conversion and provided excellent enantiomeric ratios up to >99 : 1 er. This protocol was effectively applied for the double kinetic resolution of *anti*-4,4-difluoro-1,3-diol (22b). Mechanistic studies suggested that π–cation interactions play a key role in the initial acylation of diols, stabilizing the transition state leading to the (*S*)-enantiomer by 5.42 kcal mol^−1^ relative to the (*R*)-enantiomer. In contrast, replacing fluorine atoms with chlorine reduced the reactivity due to steric hindrance. One of the diols (22d) was smoothly transformed into the valuable diamine (24) in a few steps.^37^

**Scheme 11:**
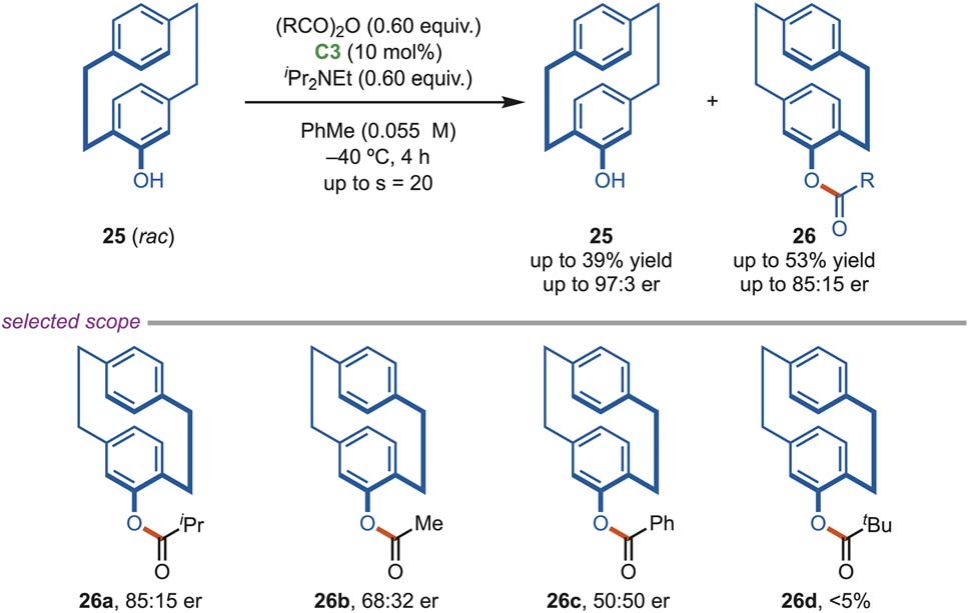
Acylative kinetic resolution of racemic cyclophane alcohols.

Planar chiral cyclophane alcohols are valuable motifs and have been applied as chiral ligands in asymmetric catalysis and as building blocks in materials and polymer chemistry. In 2021, Waser *et al.* documented an acylative kinetic resolution of racemic 4-hydroxy[2,2]cyclophane (25) using the isothiourea catalyst (Scheme 11), with a selectivity factor of up to 20. The optimal C3-catalyst showed superior activity compared to the (*R*)-C1 catalyst. Reducing the catalyst loading decreased the selectivity while maintaining a similar conversion rate. Under the optimized conditions, various anhydrides and cyclophane alcohols were tested. Notably, isobutyric anhydride produced the best results, while acid chlorides led to racemic esters (26c).^38^

**Scheme 12:**
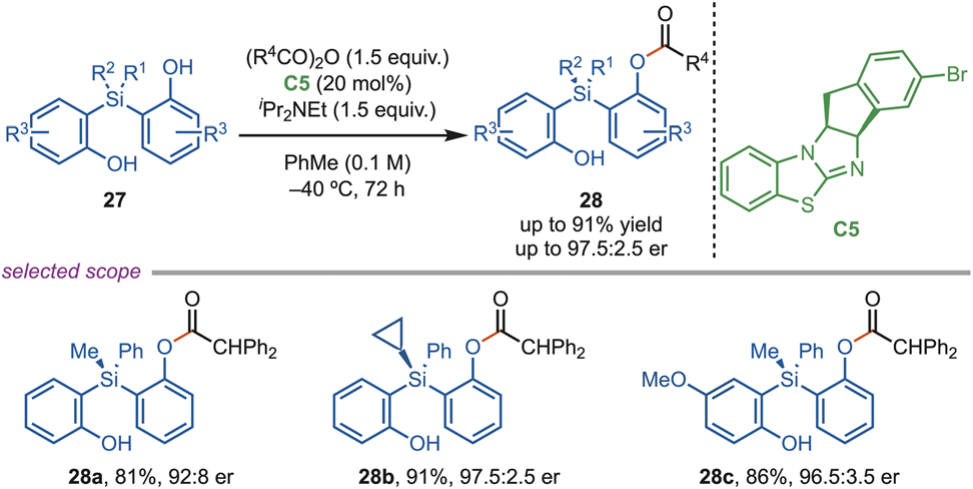
*O*-acylation of silicon-centred bisphenols.

Molecules with chiral silicon are intriguing, but their longer Si–C bonds and vacant d-orbitals result in a labile stereocenter, making selectivity more challenging to control. Despite these challenges, in 2023, Xu *et al.* introduced an isothiourea-catalyzed acylative desymmetrization strategy to construct a silicon-stereogenic centre starting from an achiral organosilane (Scheme 12). This reaction scheme involved selective monoacylation of silicon-centered bisphenols (27) with 2,2-diphenylacetic pivalic anhydride. They optimized the reaction condition at a lower temperature (−40 °C) to achieve high selectivity. The substrate scope demonstrated broad functional group tolerance, including aryl groups bearing electron-donating, electron-withdrawing substituents, halogens, heteroaryl units, and varied alkyl or cycloalkyl substituents, delivering products in moderate to excellent yields and enantioselectivities. Furthermore, the methodology was successfully scaled up to gram quantities without loss of enantioselectivity and was successfully employed to synthesize phosphine oxide derivatives.^39^

**Scheme 13:**
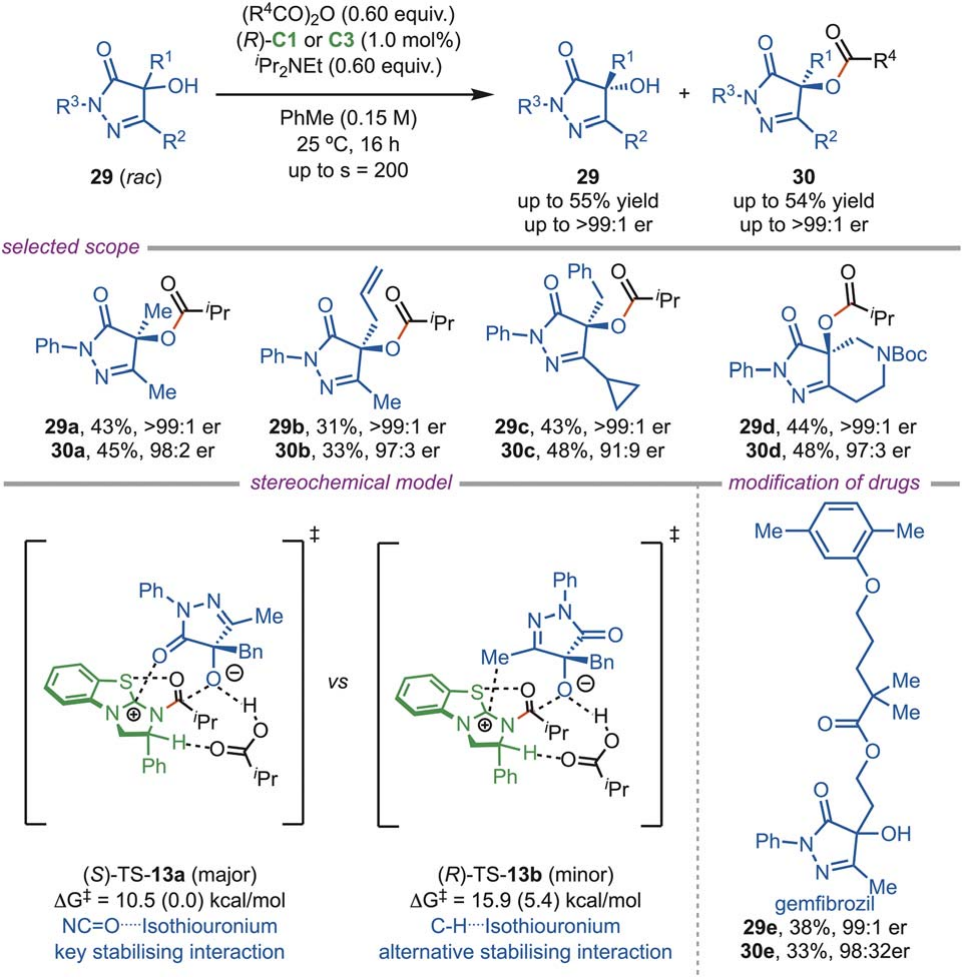
Acylative kinetic resolution of pyrazolones.

Tertiary alcohols are notoriously difficult to resolve as steric crowding creates an obstacle in chiral recognition. In 2024, the Smith group reported a novel approach to the highly enantioselective acylative kinetic resolution of racemic pyrazolone alcohol (29) (Scheme 13). With low catalyst loading, the products were formed in excellent enantiomeric excess (ee) with selectivity greater than 200. Smith's team investigated various C-4 substituents, which showed good tolerance to alkyl, propargyl, and both electron-rich and electron-poor aromatic groups. However, the phenyl substituent at the C-5 position reduced the selectivity due to competing π–isothiouronium interactions. Bicyclic and fused systems resolved well, but benzannulated structures exhibited poor selectivity. Computational studies showed that TS-13a leading to the (*S*)-isomer was favored by the stabilizing NC=O–isothiouronium interaction, whereas the other transition state was less stabilized due to the alternative π–isothiouronium interaction. The late-stage acylation of several bioactive molecules, such as gemfibrozil (29e) and probenecid, was also achieved with excellent selectivity. Effective resolution has been demonstrated on a 50 g (0.22 mol) scale, showing its practicality for industrial applications.^40^

**Scheme 14:**
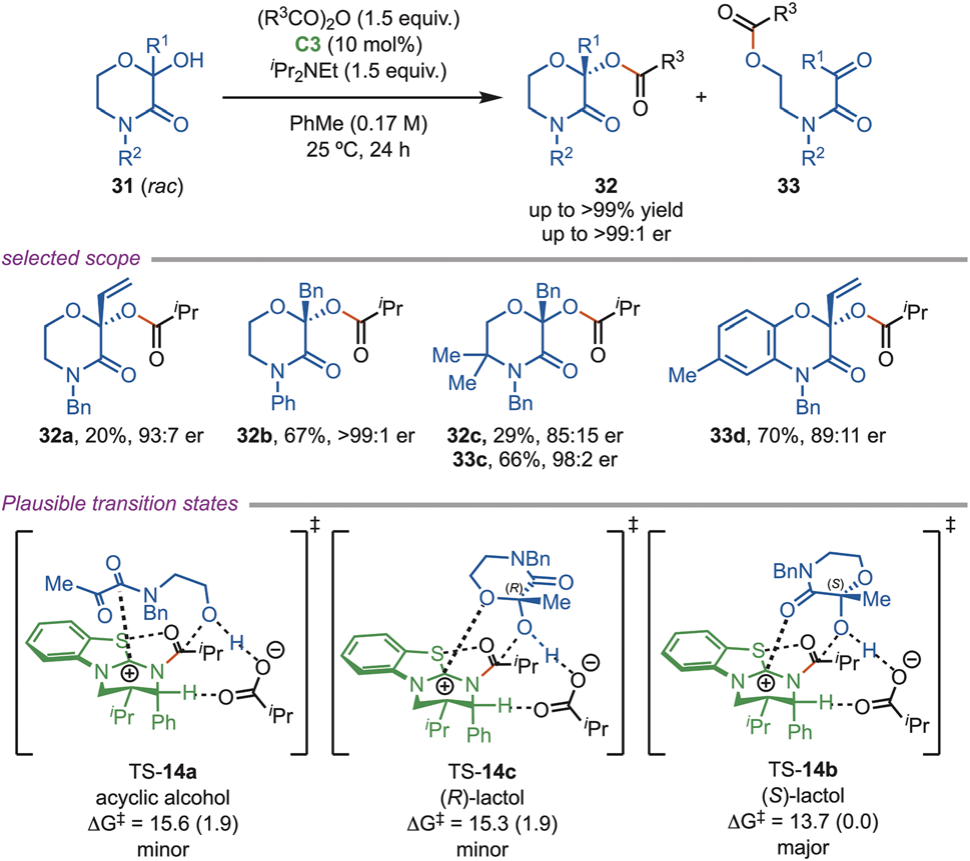
Acylative dynamic kinetic resolution of morpholine alcohols.

In the same year, Smith *et al.* introduced a dynamic kinetic resolution strategy for the enantioselective acylation of substituted morpholine and benzoxazine lactol (*rac*-31) (Scheme 14). These scaffolds are widely encountered in medicinal and agrochemical research and are present in several FDA-approved drugs, including opioid receptor ligands, antimicrobial agents, and antidepressants. This method delivered enantio-enriched products in high yields (up to 89%), with excellent enantioselectivity of up to 99 : 1 er. The C-2 substituent was generally well tolerated, although bulky or conjugated groups significantly reduced the yields of the cyclic ester. Benzoxazinone derivatives typically provided high yields, except for the C(7)-F substrate, which favored the ring-opened acylated ester in 63% yield. DFT studies indicated that ring opening and subsequent closure occurred faster than the acylation step. TS-14b gets extra stabilization arising from the N–C=O–isothiouronium interaction. Acylation of acyclic alcohol (33) was less favorable, exhibiting a higher activation barrier (15.6 kcal mol^−1^) than that of the cyclic ester (32) formation (13.7 kcal mol^−1^).^41^

**Scheme 15:**
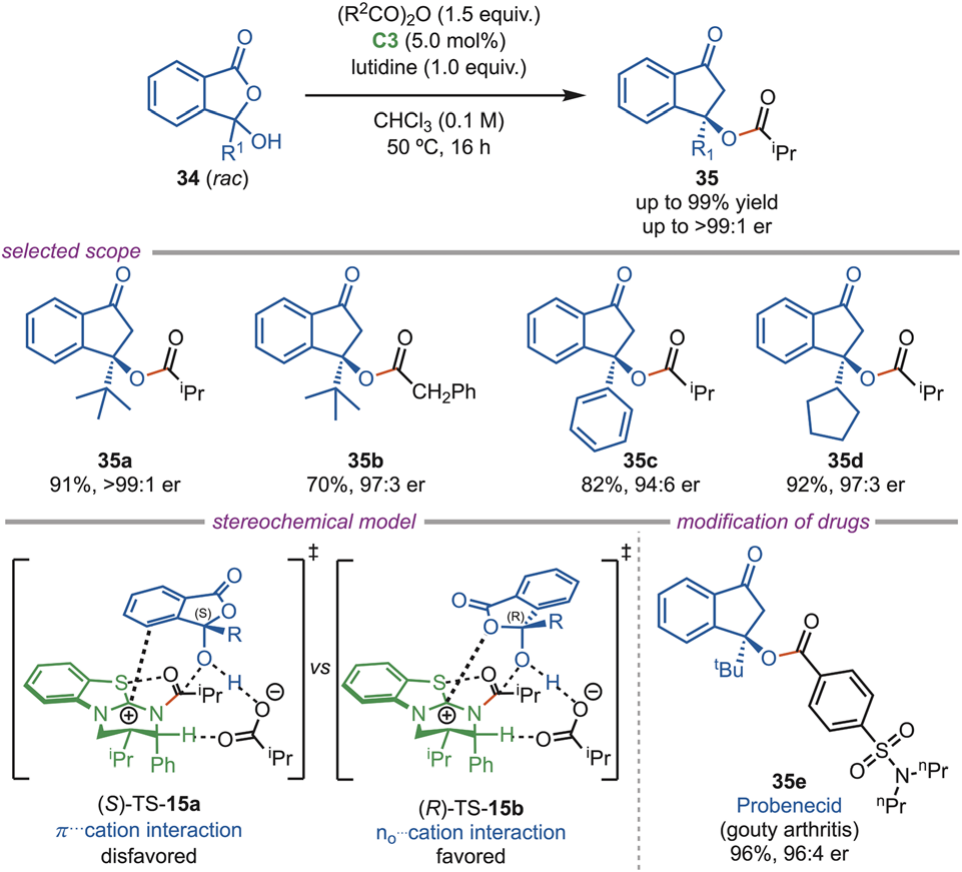
Acylative kinetic resolution and DKR of 3-hydroxyphthalides.

In 2024, the Smith group established a highly enantioselective method for synthesizing tetra-substituted 3-hydroxyphthalide esters (35) *via* dynamic kinetic resolution using an isothiourea catalyst (Scheme 15). The use of lutidine base and a 5.0 mol% C3-catalyst loading improved the enantioselectivity. This method tolerated a broad range of substrates, including linear, branched, cyclic, and aromatic C-3 substituents, and afforded products with high enantioselectivities. Diverse anhydrides, derived from aliphatic and aryl acids, were compatible. Commercial drug-containing anhydrides, such as indomethacin and probenecid, produced the acylated products in high yields with high enantioselectivity. DFT studies suggested that the (*R*)-34 enantiomer reacted faster than the (*S*)-34 enantiomer because of stronger isothiouronium interactions of C–O within the lactol and H-bonding with the anhydride, which dominated the π–cation aryl interactions in the slow-reacting TS-15a.^42^

**Scheme 16:**
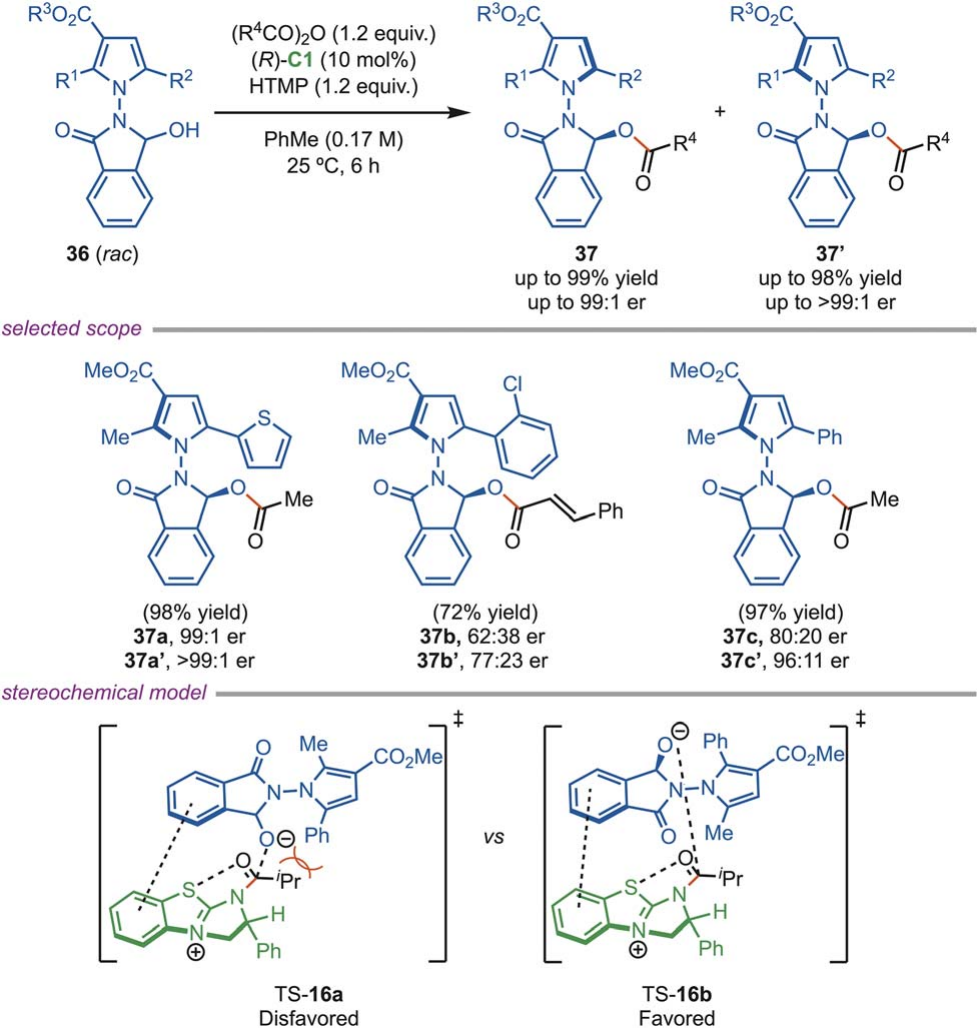
Synthesis of chiral pyrrolyl-oxoisoindolins.

In 2024, the Li group developed a novel methodology of isothiourea-catalyzed acylative dynamic kinetic resolution of hemiaminals (*rac*-36) for producing *N*–*N* axially chiral pyrrolyl-oxoisoindolins (37 and 37′) together with central chirality under mild conditions (Scheme 16). This (*R*)-C1-catalyzed dynamic kinetic resolution protocol accommodates a broad substrate scope, including various heteroaryl- and alkyl-substituted hemiaminals, with high yields and an excellent enantioselectivity of up to 99 : 1 er. TS-16b was stabilized by the π–cation and isothiouronium interactions, whereas steric repulsion destabilized TS-16a. This approach enables the efficient synthesis of *N*–*N* axially chiral pyrrolyl oxoisoindolins, which are valuable as chiral ligands and are found in drug scaffolds.^43^

**Scheme 17:**
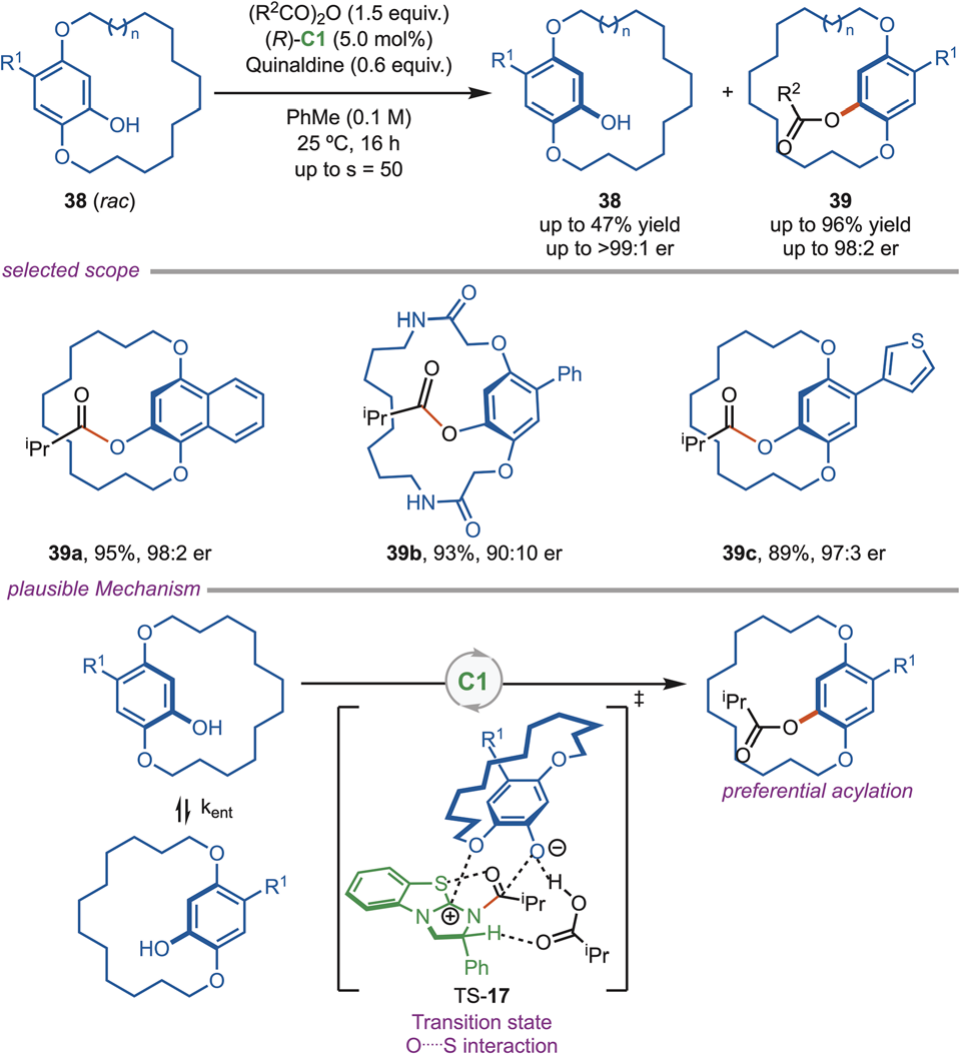
Acylative kinetic resolution and DKR of planar cyclophane alcohols using isothiourea catalysts.

In 2025, Smith and his team developed an efficient methodology for the isothiourea-catalyzed acylation of the planar-chiral cyclophane alcohol (*rac*-38) *via* kinetic resolution and dynamic kinetic resolution. They found that using 5.0 mol% of the (*R*)-C1 catalyst with isobutyric anhydride and quinaldine base was sufficient to achieve high selectivity (up to 50%) in kinetic resolution studies (Scheme 17). In the dynamic kinetic resolution, only 2.5 mol% of the (*R*)-C1 catalyst and propionic anhydride yielded high selectivity. This protocol was found to be highly selective for the 12–13-membered *ansa*-chains, where kinetic resolution operates, and the 14–18-membered chains, where dynamic kinetic resolution operates. They rationalized the observed high selectivity by hypothesizing 1,5-chalcogen bonding between the acyl oxygen and the catalyst's sulfur atom, which locked the conformation to stabilize the transition state. The transition state acted as a checkpoint: only the enantiomer that fits the catalyst's geometry passes through the lower-energy TS-17. The other enantiomer faces steric clashes, making this pathway less favorable. The significant energy difference between transition states contributes to the observed high enantioselectivity.^44^

**Scheme 18:**
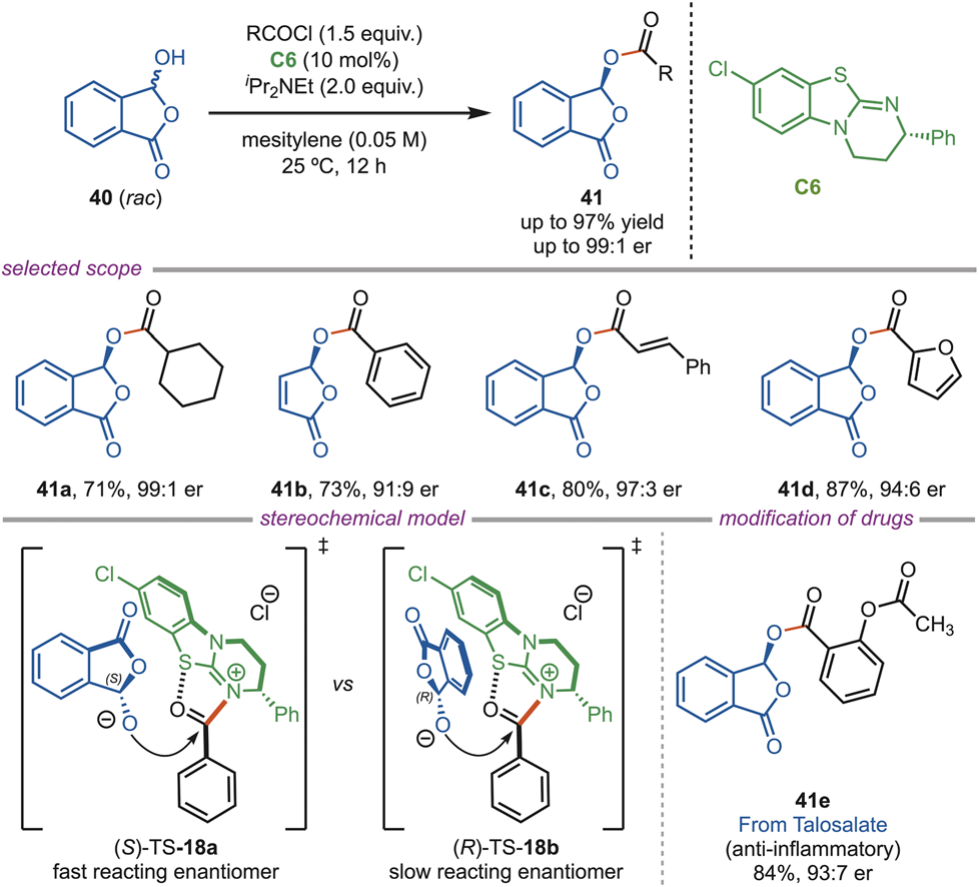
Isothiourea-catalyzed dynamic kinetic resolution of phthalides.

In 2025, Li and coworkers introduced an isothiourea-catalyzed dynamic kinetic resolution strategy for the construction of enantio-enriched phthalidyl esters (41) (Scheme 18). The substrate scope proved broad and versatile; a wide range of aryl, heteroaryl, and diverse alkyl acyl chlorides were successfully converted to the corresponding products in moderate to excellent yields and enantioselectivities (up to 99 : 1 er). The DFT study revealed that cation–π interactions and steric effects favored (*S*)-TS-18a, while the other enantiomers faced unfavorable steric clashes. Further, the robustness of the protocol was demonstrated by performing this acylation on natural products, commercial drugs, and agrochemicals.^45^

### *N*-acylation

*N*-acylation poses more challenges than *O*-acylation due to the high nucleophilicity of amines. As a result, free amines remained a challenging substrate, as they react readily with the acyl donor before the chiral catalyst can intervene, leading to poor stereoselectivity. To improve selectivity in the desired acylation reaction, it is essential to decrease the nucleophilicity of the amine while concurrently introducing steric hindrance through the installation of electron-withdrawing protective groups.^24,46–48^

**Scheme 19:**
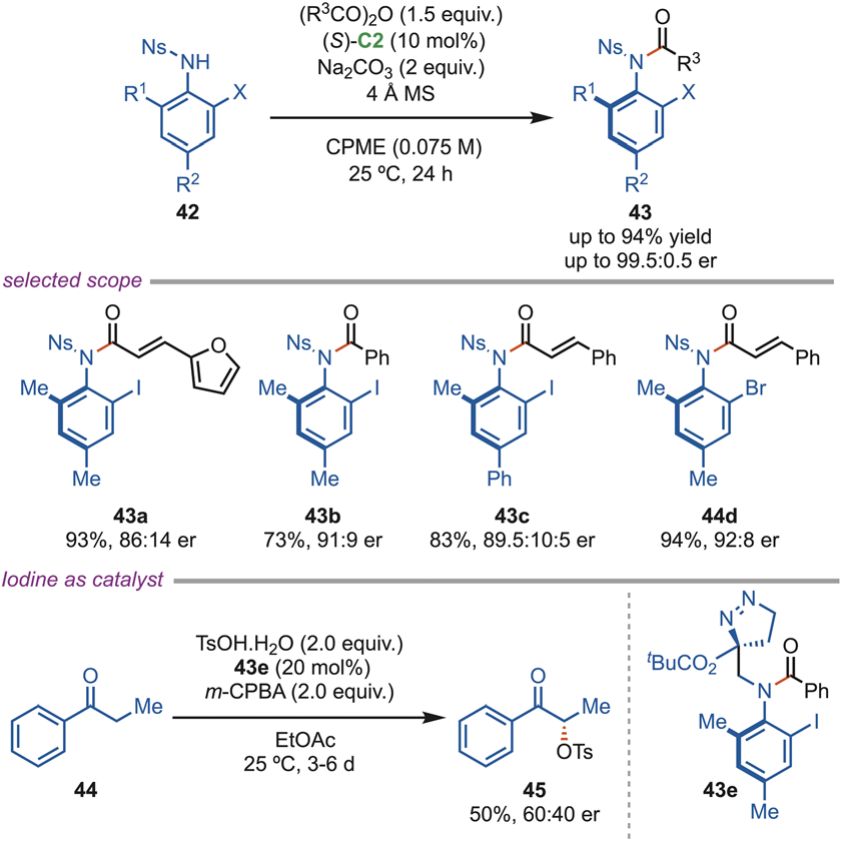
*N*-acylation of sulfonamides using isothiourea.

In 2020, the Lu and Zhao group disclosed the synthesis of axially chiral *N*-sulfonyl anilides (43) by an isothiourea-catalyzed atroposelective *N*-acylation of sulfonamides 42 (Scheme 19). The authors thoroughly investigated various reaction parameters and found that (*S*)-C2 as a catalyst, cyclopentyl methyl ether as the solvent, and 4 Å MS as additives yielded the optimal result. They demonstrated a broad substrate scope, including substituted anhydrides and sulfonamides, and the products were obtained in good yields (up to 86%) and excellent enantioselectivities (up to 99.5 : 0.5 er). They have demonstrated the usefulness of the products by employing them as a catalyst (43e) for the enantioselective α-oxytosylation of propiophenone (44), and the corresponding product (45) was obtained in moderate yield and enantioselectivity.^49^

**Scheme 20:**
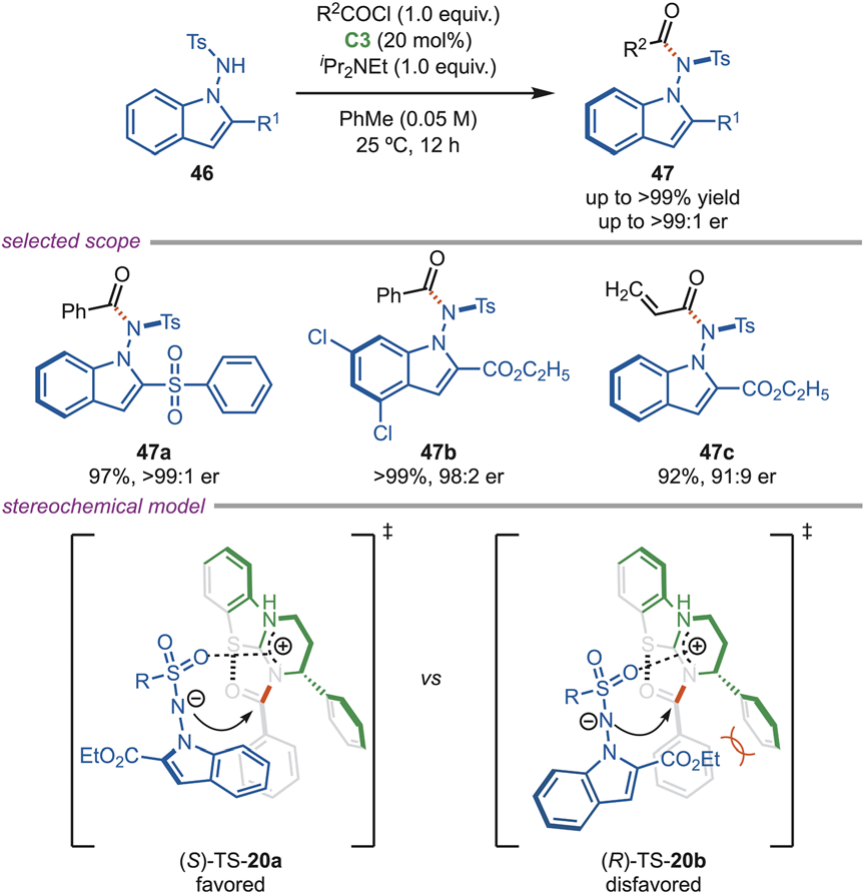
Atroposelective *N*-acylation of *N*-aminoindoles.

In 2024, Gan and Chi *et al.* developed an asymmetric atroposelective acylation reaction for the synthesis of *N*–*N* axially chiral indolylamides (47) with acyl chloride as an acylating agent (Scheme 20). This reaction proceeds *via* dynamic kinetic resolution of *N*-acylaminoindoles using the C3-catalyst, and the axially chiral products were obtained in excellent enantiomeric excess. *N*-aminoindoles with diverse substituents on the indole ring, as well as on the aryl sulfonyl ring, were obtained in excellent er. The steric repulsion between the ester group on the indole and phenyl substituents on the C3-catalyst makes the (*R*)-TS-20a less favorable, and the (*S*)-enantiomer is formed as the major product. One of the *N*–*N* axially chiral molecules shows excellent antibacterial activity against *Xanthomonas axonopodis pv. citri* (*Xac*) bacteria.^50^

**Scheme 21:**
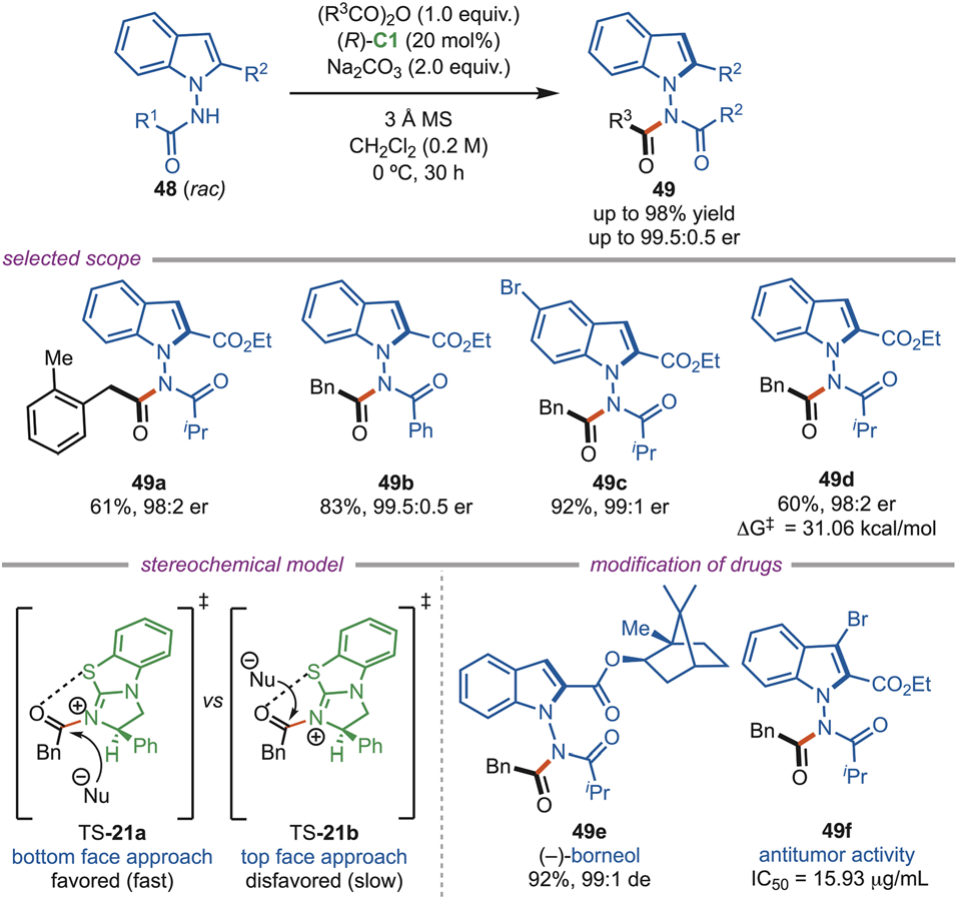
Atroposelective *N*-acylation of *N*-aminoindoles.

In the same year, Zhang and Shi *et al.* also developed an asymmetric atroposelective acylation reaction for the synthesis of *N*–*N* axially chiral indolylamides (49) with anhydride as the acylating agent (Scheme 21). The substrate scope includes diverse substitution patterns on both the partners, and the products were formed in good to excellent yield and enantioselectivities (up to >99 : 1 er). The transition state model demonstrated that the acyl ammonium intermediate arranged the reacting groups in a precise three-dimensional configuration. TS-21a was stabilized by *syn*-coplanar 1,5-S⋯O interactions, allowing one atropoisomer to react smoothly while the other form was hindered. One of the synthesized compounds (49f) exhibited significant antitumor activity against the HepG2 cancer cell line.^51^

**Scheme 22:**
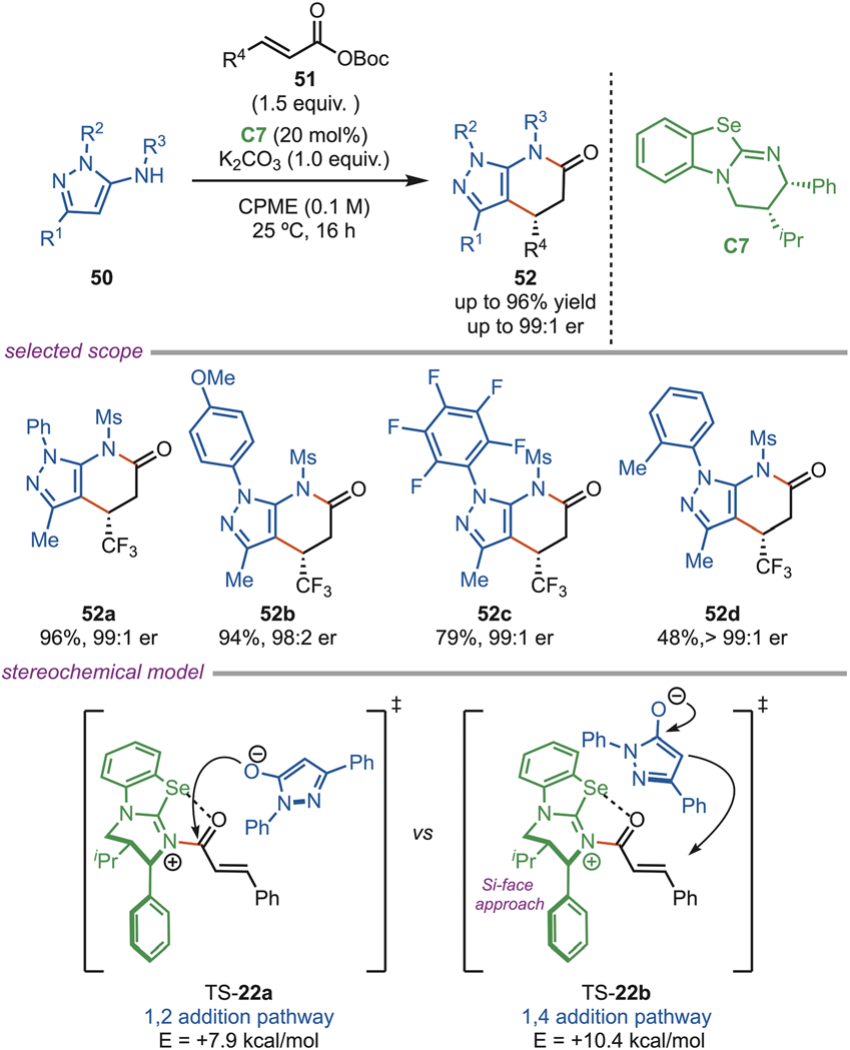
Enantioselective synthesis of fused pyrazoles.

Nitrogenous heterocycles, such as fused pyrazoles, are valuable scaffolds commonly used in the pharmaceutical and agrochemical industries. In 2025, the Bühl and Smith group reported an enantioselective synthesis of fused pyrazolo-heterocycles (52), which was achieved *via* a [3 + 3] Michael addition–cyclization pathway that operates *via* a self-correcting amide and ester acylation mechanism (Scheme 22). Further, isothiourea C3 was not limited to serving as a catalyst; they also used isoselenourea as a catalyst to deliver products with excellent yields and enantioselectivity of up to >99 : 1 er. The DFT studies revealed that isoselenourea (C7) showed superior performance over the C3-catalyst because the acylated intermediate was stabilized by *n*(O) → σ*(Se–C) interaction and exhibited higher reactivity. The twisted amide geometry (10.5° Winkler–Dunitz angle) allows for reversible acylation and with a unique self-correcting pathway.^52^

## Conclusions

In conclusion, the advancements in asymmetric acylation reactions over the past decade demonstrate the significant potential of chiral isothiourea catalysts for the synthesis of enantioenriched alcohols and amines. The pioneering work of Birman and subsequent contributions from various research groups, particularly the Smith group, have established a solid foundation for kinetic resolution and dynamic kinetic resolution methodologies applicable to racemic heteroatom-containing substrates. Notably, the development of recyclable polymer-supported immobilized catalysts operating at low loadings represents a promising avenue for large-scale industrial applications in the production of chiral building blocks. Furthermore, insights from DFT calculations highlight the critical roles of isothiouronium interactions and secondary stabilization factors in achieving high enantiomeric ratios. These insights could be used to design the next generation of isothiourea catalysts, either by introducing high-valent sulfur or selenium to enhance the *n*(O) → σ* interaction, or by utilizing gem-diaryl groups on the HBTM derivative to introduce additional π–interactions. However, while significant strides have been made in the synthesis of chiral alcohols, the relatively limited exploration of asymmetric *N*-acylation reactions for racemic amines presents an exciting opportunity for future research. Given the prevalence of chiral amines in natural products and pharmaceuticals, addressing this gap could lead to further innovations in the field. Continued exploration of these synthetic strategies will be essential to harness the full potential of chiral isothiourea catalysts across a broader range of applications.

## Author contributions

S. A. K. and A. K. S. decided on the topic. S. A. K. did the literature review and prepared the first draft of the review. R. G. helped write the *N*-acylation section and proofread. A. K. S. supervised and completed the project.

## Conflicts of interest

There are no conflicts to declare.

## Data Availability

No primary research results, software or code have been included and no new data were generated or analysed as part of this review.
